# Supplementary material for: Chromothripsis during telomere crisis is independent of NHEJ, and consistent with a replicative origin
Source: Genome Res. 2019 May;29(5):737–49. doi: 10.1101/gr.240705.118 (PMC6499312; doi:10.1101/gr.240705.118)
Supplement: Supplemental Material [file supp_gr.240705.118_Supplemental_file_1.zip › contigs/annotated_contigs/DB51/contig.2.DB51_length_282_mean_cov_6.91489361702.docx]

**DB51_length_282_mean_cov_6.91489361702**

```
TCACCCACGTGCGTGGTTTTACCCCGTCTCCATGACCCAGCCACGGGTCTTATATTGGGGGCTTCTAGGACCAACTAGGCCAATAGAAG
 >chr6:1683102-1683264 - E=2e-86 p=1e-02
TGCTTTCTTTAATTGAATTAAAATGTCAGTAAATTTCACCCAAATATCCAGATTTCCCTTTCT|AAAAAAATGA|AAGGCCATCAGCAC
                                                                >chr6:1682671-1682801 -
TGGGGCAGATTCCTCACTGACTGGCCTGCCCGCCTGCCTGGCCTAGTGGGCACACAGAGTCCCCGGTCCTCCCAGTGGCAATGGCACCA
 E=5e-67
GCATGGCCAGAGGGCAC
```
